# Characterisation of the proximal airway squamous metaplasia induced by chronic tobacco smoke exposure in spontaneously hypertensive rats

**DOI:** 10.1186/1465-9921-10-118

**Published:** 2009-11-24

**Authors:** Sarah J Bolton, Kate Pinnion, Victor Oreffo, Martyn Foster, Kent E Pinkerton

**Affiliations:** 1 Safety Assessment UK, AstraZeneca R&D Charnwood, Bakewell Road, Loughborough, Leicestershire, LE11 5RH, UK; 2 Centre for Health and & Environment, University of California, Davis, CA 95616, USA

## Abstract

**Background:**

Continuous exposure to tobacco smoke (TS) is a key cause of chronic obstructive pulmonary disease (COPD), a complex multifactorial disease that is difficult to model in rodents. The spontaneously hypertensive (SH) rat exhibits several COPD-associated co-morbidities such as hypertension and increased coagulation. We have investigated whether SH rats are a more appropriate animal paradigm of COPD.

**Methods:**

SH rats were exposed to TS for 6 hours/day, 3 days/week for 14 weeks, and the lung tissues examined by immunohistochemistry.

**Results:**

TS induced a CK13-positive squamous metaplasia in proximal airways, which also stained for Ki67 and p63. We hypothesise that this lesion arises by basal cell proliferation, which differentiates to a squamous cell phenotype. Differences in staining profiles for the functional markers CC10 and surfactant D, but not phospho-p38, indicated loss of ability to function appropriately as secretory cells. Within the parenchyma, there were also differences in the staining profiles for CC10 and surfactant D, indicating a possible attempt to compensate for losses in proximal airways. In human COPD sections, areas of CK13-positive squamous metaplasia showed sporadic p63 staining, suggesting that unlike the rat, this is not a basal cell-driven lesion.

**Conclusion:**

This study demonstrates that although proximal airway metaplasia in rat and human are both CK13+ and therefore squamous, they potentially arise by different mechanisms.

## Background

Chronic obstructive pulmonary disease (COPD) is characterised pathologically by loss of lung elasticity, airspace enlargement, small airway remodelling and inflammation [1]. It is widely acknowledged that tobacco smoke (TS) is linked to the development of chronic obstructive pulmonary disease (COPD) in humans. The epithelial mucosa of the lung is the primary site of initial exposure to TS. Repeated cycles of damage and repair to this mucosa in response to chronic TS exposure can result in bronchial epithelial squamous metaplasia, a histopathological feature of COPD, particularly in moderate to severe disease [2,3]. Squamous metaplasia of the airways is seen as a rapid repair mechanism akin to wound healing to maintain barrier integrity, that is reversible given appropriate conditions, and mediates restitution of the normal airway phenotype [4]. Normal pulmonary (bronchial) epithelial repair mechanisms in response to injury involve the dedifferentiation of epithelial cells to produce a squamous cell covering that maintains mucosal integrity. The epithelium is then repopulated via resident basal cell proliferation, which differentiate to form a new mature epithelial barrier [5]. Repeated insults such as continued smoking, or a delay in the differentiation and maturation of the epithelium can result in squamous metaplasia that becomes irreversible. Recent evidence by Araya and co-workers [6] indicates that areas of squamous metaplasia are in communication with the underlying mesenchyme, and via activation of TGFβ, results in fibrosis and small airway wall thickening. Thus, the presence of squamous metaplasia has important pathological consequences.

There are a variety of markers that reflect the particular status or differentiated state of an epithelial cell. For example, cytokeratins (CKs) have been widely used to distinguish between different types of pulmonary epithelial cells [7,8] and in humans are used to differentiate between different types of lung carcinomas and sarcomas [9]. There is a good level of homology between human and rat CKs [10] and this has also been shown in rat bronchial carcinomas [11]. In particular, CK13 is a marker for well-differentiated squamous cell carcinoma in rats and humans. The transcription factor p63 is a homologue of the p53 tumour suppressor protein and is considered as reliable a marker of basal cells as high molecular weight cytokeratins [12]. Factor p63 is proposed to be important in the maintenance of epithelium stem cell populations and is expressed on basal epithelial cells from many organs including the lung [13,14]. Factor p63 exists in 2 alternatively transcribed isoforms: either a full length transcript (transactivating or TAp63) or with deletion of the TA domain (truncated or ΔNp63). The function of the 2 isoforms are different as TAp63 functions similar to p53 and promotes cell cycle arrest and apoptosis whereas the ΔNp63 isoform is predominantly expressed in proliferative epithelial stem cell populations and can inhibit the p53-like functions of p63TA. The ΔNp63 isoform shows homology with a number of recently identified transcription factors that are all specifically expressed in squamous cell carcinomas [15-17]. Thus, ΔNp63 appears to play a key role in the development of a squamous cell phenotype.

Rodent bronchial epithelial cells have a very rapid turnover rates compared to humans and therefore lesions tend to resolve quickly and spontaneous squamous metaplasia is rare in rodents [18]. Squamous metaplasia can be induced in rodents in response to various agents such as TS [19], dioxins [20] or mineral dusts [21], although bronchial neoplasias are difficult to induce as most of the pathology occurs more peripherally within the lung parenchyma. Recently, Zhong and co-workers [22] described the presence of squamous metaplasia in the proximal airways following chronic TS exposure in spontaneously hypertensive (SH) rats. These SH rats are known to be more susceptible to airway disease compared to non-SH rats [23]. For example, when SH rats are exposed to sulphur dioxide for 5 days, they develop bronchitis that is characterised by a neutrophilic inflammation and mucus hypersecretion [24]. Also, acute exposure to TS will induce a more robust inflammatory infiltrate compared to Wistar-Kyoto rats [25]. SH rats are known to share some key underlying pathologies with human COPD including borderline hypertension, a hypercoagulative state and oxidative stress [23,24]. SH rats have a defective CD36 scavenger receptor [26], but the role for this in respiratory processes is unclear. There is now an accumulating body of data that suggest that the SH rat, due to its shared physiological characteristics with COPD patients, is a more appropriate strain of rat in which to model airway disease.

In this study, we describe the phenotype of the epithelial response in the lung to chronic TS exposure in SH rats. We have examined, using an immunohistochemical approach, the molecular profile of the epithelial cells in both the proximal (bronchi and bronchioles) and distal (respiratory bronchioles) airways and within the alveolar bed. We have also compared the p63 profile seen in the TS exposed rats with samples from human COPD lung and the data suggest that despite morphological similarities, squamous metaplasia arises via different mechanisms in humans and rodents following chronic TS exposure.

## Methods

### Antibodies and reagents

See Table 1 for details of antibodies and their detection systems. All other reagents were of analytical grade.

**Table 1:** Antibodies used to characterise the epithelial response to TS in SH rats.

Antigen	Source	**Catalogue No**.	Clone and Isotype	Conc, μg/ml	Antigen Retrieval	Detection System
Caspase 3, activated	Cell Signalling	9661	Rabbit IgG	1.0	MW, citrate	StreptABC HRP, DAB

CC10	Cell Signalling	07-623	Rabbit IgG	1.0	MW, VUF	StreptABC HRP, DAB

CD31	Serotec	MCA1334G	TLD-3A12, IgG1	6.67	BA	TSA kit, HRP, DAB

Ki67	Vector	VP-K452	MM1, IgG1	0.5	PC, VUF	TSA kit, HRP, DAB

CK13	Chemicon	CBL176	Ks13.1, IgG1	0.5	PC, VUF	Envision HRP, DAB

Pan CK (4,5,6,8,10,13,18)	Chemicon	Mab 1636	C11, IgG1	5.0	MW, VUF	StreptABC HRP, DAB

p40	Calbiochem	PC373	Rabbit IgG	1/2000 diln	MW, citrate	StreptABC HRP, DAB

p63	Pharmingen	559951	4A4, IgG1	5	None	StreptABC HRP, DAB

Phospho-p38	Santa Cruz	17852	Rabbit IgG	1	None	TSA kit, HRP, DAB

Surfactant D	Abcam	AB15687	SPDE, IgG2b	5	PC, VUF	TSA kit, HRP, DAB

MW = Microwave; PC = pressure cook; BA = 0.2 M boric acid, pH7.0, 60°C 16-18 hours;VUF = Vector Unmasking Fluid (citrate = 0.01 M Na citrate, pH6.0);

StreptABC kit from Dako, used according to manufacturer's instructions; TSA kit from Perkin Elmer, used according to manufacturer's instructions; HRP HorseRadish Peroxidase; DAB = diaminobenzidine.

### Exposure of rats to TS and necropsy

The rat lung sections used in this study were taken from rats as described previously [22]. Briefly, 12 week old SH rats (260-310 g) were purchased from Charles River Laboratories (Raleigh, NC, USA) and allowed to acclimatise for 1 week. They were maintained on a 12 hour light/dark cycle and provided with water and rat chow *ad libitum*. Animals were handled in accordance with standards established by the US Animal Welfare Acts as set forth in the National Institutes of Health guidelines and by the University of California, Davis, Animal Care and Use Committee. Rats were exposed to a mixture of mainstream and sidestream smoke from humidified 1R4F cigarettes (Tobacco Health Research Institute, Lexington, KY, USA). An automatic metered puffer was used to smoke the cigarettes under Federal Trade Commission conditions (35 ml/puff, 2s duration, 1 puff/min). The smoke was collected via a chimney and delivered to whole body chambers. The animals were exposed to either filtered air (FA) or high concentrations of TS (80 mg/m^3^) for 6 h/day, 3 days/week for either 7 or 14 weeks. At necropsy, each animal was given an overdose of sodium pentobarbital. The trachea was cannulated and the left lung lobe was inflation-fixed by intra-tracheal instillation of 4% buffered zinc formalin (Z-fix) at 30 cm water pressure for 1 hr and stored in 70% ethanol before processing. Transverse slices were taken immediately cranial and caudal to the hilum of the lobe, dehydrated in a graded ethanol series and embedded in paraffin wax.

### Human subjects and tissue

Human lung samples were obtained with written informed consent from patients undergoing lung volume reduction surgery at the Glenfield Hospital, Leicester. Patients (GOLD4) were selected according to the inclusion criteria described previously [27]. Tissue was fixed in 10% neutral buffered formalin (NBF) within 2-3 hours of surgery, and was fixed for a further 48 hours in 10% NBF. The tissue was embedded in paraffin using standard procedures. Fourteen blocks from 8 different patients were used in this evaluation.

### Immunohistochemistry

Sections from rats (5 per group) exposed to either FA or high concentration of TS (80 mg/m^3^) were used in this study as previously described [22]. 5 μm sections were cut on a microtome and picked up on charged slides and dried overnight at 37°C. Sections were dewaxed in xylene, taken through graded alcohols into water. For staining with haematoxylin and eosin (H&E), sections were stained with Gills II haematoxylin (Pioneer Research Chemicals, Colchester, Essex, UK) and eosin Y (Acros Organics, Fisher Scientific, Loughborough, Leicestershire, UK) on a Leica ST5020 Autostainer (Leica Microsystems, Milton Keynes, Buckinghamshire, UK). Antigen retrieval was performed as detailed in Table 1. Heat treatment was by microwave (98°C, 5 mins, RHS-2 rapid Microwave Histoprocessor, Milestone Srl, Sorisole, Italy) or pressure cooker (Prestige, 15l bs, 2 mins). Sodium citrate buffer was prepared to pH6 and Vector Unmasking Fluid (Vector Labs, Peterborough, Cambs, UK) was used according to manufacturer's instructions. Boric acid retrieval has been described before [28]: sections are incubated in 0.1 M boric acid, 60°C for 16 hrs. All steps were carried out using a LabVision Autostainer except for incubation in the chromogen diaminobenzidene (DAB). Bound antibody was detected using either standard Streptavidin-Biotin complex protocols (StreptABComplex, Dako), TSA amplification kit (Perkin Elmer) or Envision polymer technology (DAKO) according to manufacturer's instructions (see Table 1). Sections were then counterstained in Gills II Haematoxylin, dehydrated and mounted in DPX mounting medium (Merck, Lutterworth, Leicestershire, UK).

## Results

### Morphology of TS induced changes in proximal and distal airways of SH rats

**Figure 1:**
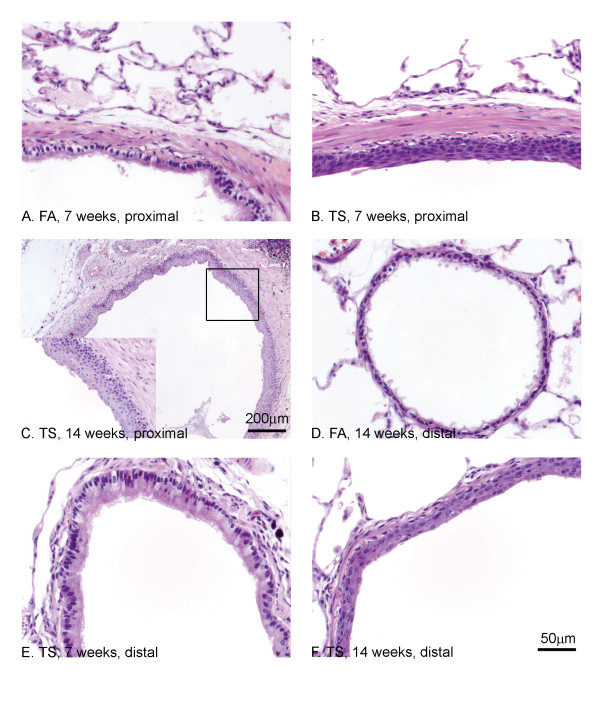
**Morphology of TS induced changes in proximal and distal airways of SH rats**. H&E stain for pathological assessment. Morphological changes were seen in proximal (A-C) and distal airways (D-F) following TS exposure. FA exposed rats showed minimal changes to proximal (A) and distal (D) airways. Exposure to TS for 7 (B) and 14 (C) weeks induced a squamous metaplasia in proximal airways (see high power inset in panel C). Distal airways showed epithelial hypertrophy at 7 weeks (E) but progressed to squamous metaplasia in occasional airways by 14 weeks (F). Magnification bar in F applies to panels A, B, D-F.

Rats exposed to FA for 7 or 14 weeks showed minimal changes, mainly rarefaction of epithelial cell cytoplasm and sporadic hypertrophy (Fig. 1A). Exposure to high concentrations of TS (80 mg/m^3)^, led to the induction of a squamous metaplasia in the proximal airway (bronchi and bronchioles) at 7 and 14 weeks as has been described previously [22]. At 7 weeks, the normal respiratory epithelium was replaced by metaplastic squamous epithelial cells with occasional stratification (Fig. 1B). By 14 weeks the entire proximal airway was composed of stratified, keratinizing squamous epithelial cells (Fig. 1C). Compared to the FA controls (Fig. 1D), the smaller distal airways (respiratory bronchioles) showed epithelial hypertrophy and hyperplasia at 7 weeks (Fig 1E) After 14 weeks exposure, most distal airways were hyperplastic but in three out of five rats, there were occasional airways showing squamous metaplasia, although this was not as advanced as in the proximal airways and was largely composed of just 1 or 2 layers of cells (Fig. 1F).

### Morphology of TS induced changes in the lung parenchyma of SH rats

**Figure 2:**
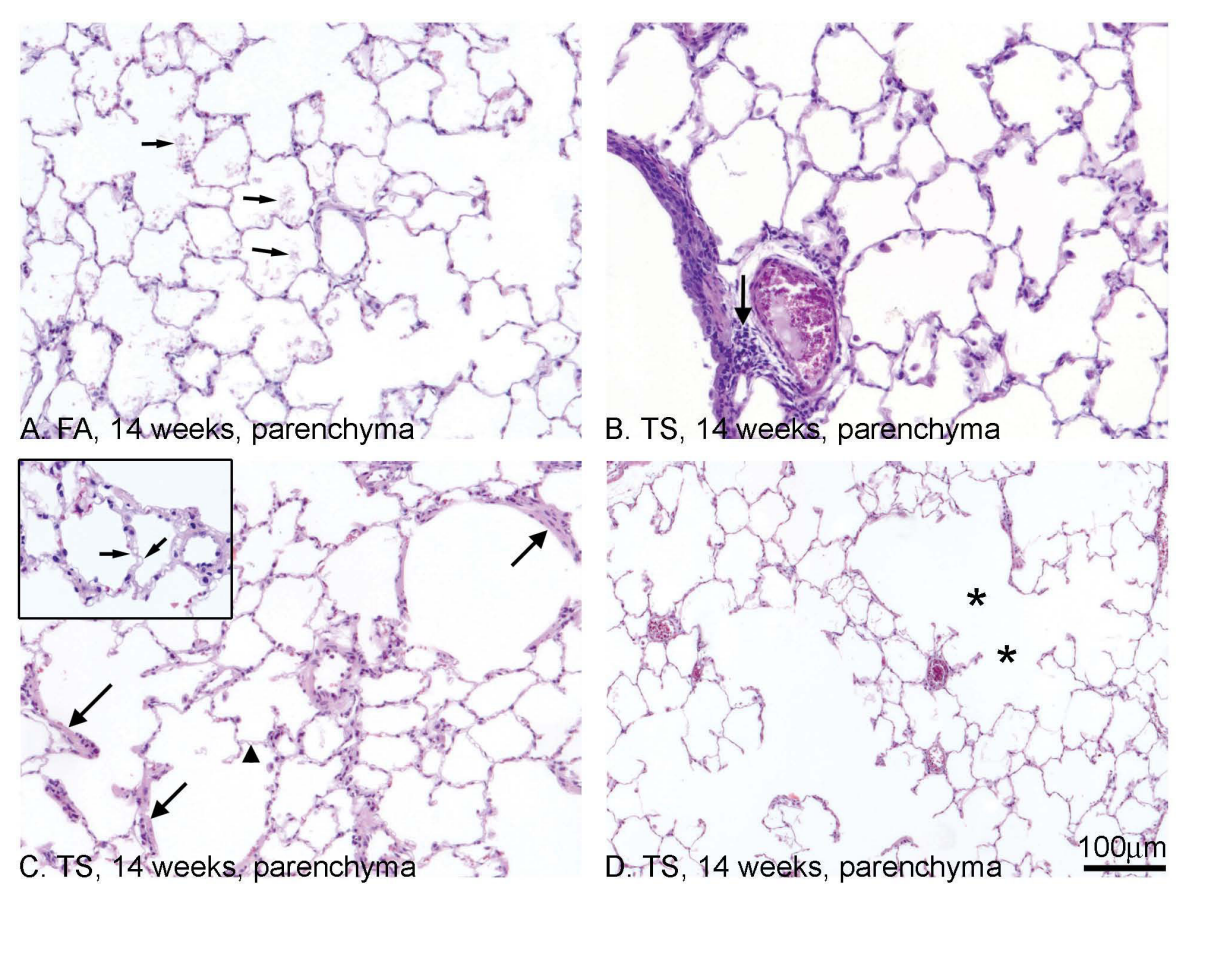
**Morphology of TS induced changes in the lung parenchyma of SH rats**. H&E stain for pathological assessment. FA exposed rats showed fibrinoid leakage from the alveolar bed (A, arrows) but minimal changes to resident cells. Following TS exposure for 14 week, there was evidence of an inflammatory infiltrate within perivascular/peribronchiolar regions (B, arrow). There was also fibrosis of blood vessels and the alveolar bed (C, arrows). Casts of the alveolar capillaries (C, inset, arrows) indicated remodelling of the microvascular network. Loss of connectivity was also seen (D, asterisks) within the alveolar bed.

Rats exposed to FA for 7 or 14 weeks showed minimal changes, mainly a low-grade alveolitis (Fig. 2), including fibrinoid microvascular leakage (Fig. 2A, arrows). Following TS exposure, changes seen in the parenchyma at 7 weeks appeared to progress in severity and incidence by 14 weeks. At 7 weeks, there was a macrophagic inflammation including the presence of foam cells in the alveolar bed and perivascular/peribronchiolar leucocytes (Fig. 2B, arrow). The inflammation was reduced at 14 weeks but there were now signs of type II pneumocyte proliferation and remodelling of the parenchyma including mesenchymal expansion of the alveolar walls and perivascular regions typical of fibrosis (Fig. 2C, arrows). In addition, there was evidence of remodelling (cast formation) of the microvasculature in the alveolar bed (Fig. 2C inset, arrows), hyperinflation and enlarged airspaces, as well as loss of connectivity within the parenchyma (Fig. 2D, asterisk).

### Cell turnover following TS exposure in SH rats

**Figure 3:**
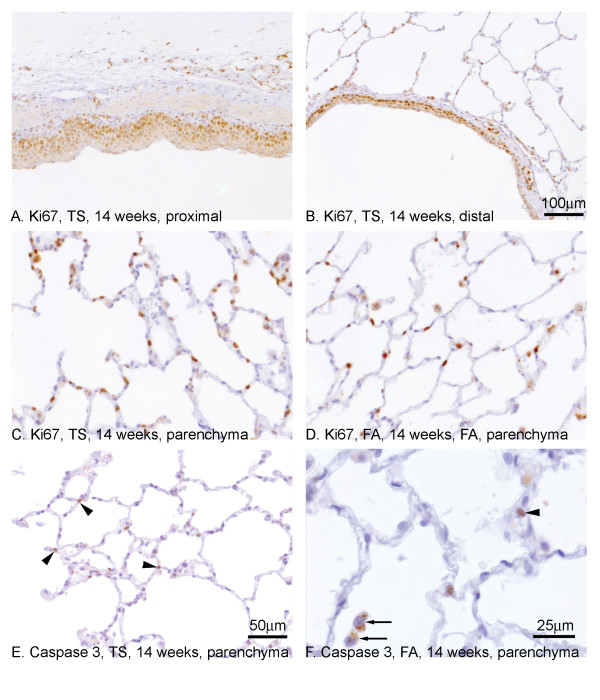
**Cellular turnover following TS exposure in SH rats**. Antibodies to Ki67 (A-D) and cleaved caspase 3 (E, F) were used to determine the level of cell turnover following TS exposure. Areas of squamous metaplasia in both proximal (A) and distal (B) airways were strongly and extensively stained for Ki67 compared to FA controls (data not shown). Within the alveolar bed, the numbers of cells staining for Ki67 was increased after TS exposure (C) compared to the FA controls (D). Staining for cleaved capsase 3 showed a marginal increase in the numbers of cells staining following TS exposure (E, arrowheads) compared to FA controls (F, arrowhead). Staining was also seen in vesicles of alveolar macrophages (F, arrows). Magnification bar in B applies to panels A and B. Magnification bar in E applies to panels C-E.

In order to investigate the balance between cell proliferation and cell death via apoptosis, sections were immunostained with either anti-Ki67 antibodies (proliferation) or anti-activated caspase 3 antibodies (apoptosis). In FA exposed rats, staining for Ki67 was sporadic showing occasional nuclear staining throughout all compartments of the lung at both 7 and 14 weeks (data not shown). In the TS exposed rats, within the area of squamous metaplasia, all of the cell nuclei were positive for Ki67 regardless of the site or time of exposure (Fig. 3A, proximal airway; Fig. 3B, distal airway, both 14 weeks). Within the smaller non-metaplastic bronchioles there was an apparent increased incidence of staining in epithelial cells following TS exposure (data not shown). In TS exposed rats, Ki67 staining was seen in the parenchyma, particularly of cells judged on their morphology to be type II pneumocytes (Fig. 3C, 14 weeks). There appeared to be fewer numbers of cells staining in the FA exposed rats (Fig. 3D).

Previously, Zhong et al., have documented the presence of TUNEL-positive apoptotic cells in proximal, distal and parenchymal cells in the same model and shown increases in the numbers of TUNEL-positive cells in main proximal and distal bronchioles but not in the parenchyma [29]. Using an anti-activated caspase 3 antibody, we found only sporadic staining within the airway epithelial cells of either the proximal or distal airways (data not shown). However, staining was more apparent within the parenchyma, particularly in the nucleus of cells with a morphology typical of Type II pneumocytes, following TS exposure at both time points (Fig. 3E, 14 weeks, arrowheads). The data indicated a slight increase that was more apparent at 7 weeks. Caspase 3 staining was also seen in vesicles typical of the lysosomal compartment of alveolar macrophages (Fig. 3F, arrows).

### Epithelial profile of TS induced squamous metaplasia in proximal airways

**Figure 4:**
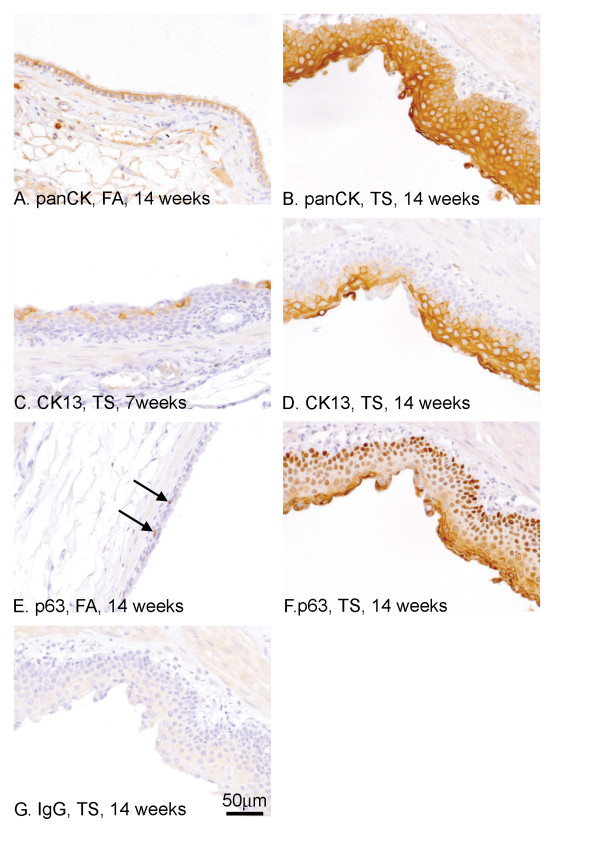
**Epithelial profile of TS induced squamous metaplasia in proximal airways**. Antibodies against pan CK (A, B), CK13 (C, D) and p63 (E, F, G) were used to characterise the squamous metaplastia seen in proximal airways following TS exposure. All epithelial cells were positive for panCK (A, B). After 7 weeks TS exposure, there was sporadic staining in areas of squamous metaplasia (C) but after 14 weeks, TS exposed rats showed CK13-positive staining in suprabasal regions of the entire airway (D). Staining for p63 highlighted sporadic basal stem cells in FA exposed rats (E, arrow). After 14 weeks of TS exposure in larger airways, all cell nuclei in areas of squamous metaplasia (F) were stained but with a gradation of intensity from the basal cells (intense) to the suprabasal cells (weak). Staining with the isotype control antibody (G) showed no specific nuclear staining although there was weak background staining in the cytoplasm.

We used antibodies to generate a molecular profile of the epithelial cells following TS exposure. An anti-pan CK (which recognises CKs 4, 5, 6, 8, 10, 13, 18) was used to highlight all epithelial cells. Epithelial cells in all airways, regardless of treatment, were positively stained (Fig. 4A, FA, 14 weeks). The squamous metaplasia seen at either 7 or 14 weeks was very strongly stained (Fig. 4B, TS, 14 weeks). In addition to the airways, sporadic cells within the alveolar bed were positively stained and, from their morphology, were presumed to be type II pneumocytes (data not shown). CK13 was used to highlight the mature squamous epithelial in the areas of metaplasia. There was no staining in FA exposed rats as expected (data not shown) but after 7 weeks TS exposure, there was sporadic staining in the proximal airways (Fig. 4C) but by 14 weeks, cells in the parabasal or superficial layers of the squamous metaplasia were strongly positive (Fig. 4D). Factor p63 is a marker of basal epithelial cells and normally shows nuclear staining of flattened basal cells (Fig. 4E, arrow). Following TS exposure, there was a marked increase in the numbers of positive cells in proximal airways in areas of squamous metaplasia. Staining was restricted to nuclei of the basal and parabasal cells where it showed a graded intensity, which decreased with distance away from the basement membrane (Fig. 4F, 14 weeks). Staining was also seen in the acellular keratotic layer of the metaplasia but the reason for this is unclear as an isotype control incubated section did not show this staining pattern (Fig. 4G). No staining was seen in the smaller distal airways without pathology or the parenchyma. We further investigated the role of p63 by immunostaining for the ΔNp63 truncated isoform using the p40 antibody [15]. Staining was identical to the p63 pattern and was restricted to the nuclei of basal and parabasal cells of the squamous metaplasia (data not shown).

### Association of functional markers with squamous metaplasia in proximal and distal airways

We investigated whether the conversion of normal respiratory epithelium to squamous epithelial cells would alter the profile of some functional markers. We used CC10 as a marker for Clara cells, which are known to respond to inhaled toxicants [30-32] and has been proposed as a peripheral biomarker of airway disease. We have previously shown that expression of CC10 and surfactant protein D (SP-D, expressed in Clara cells and a marker of type II pneumocytes) can be induced in previously negative cell types within the alveolar bed in response to an inflammatory stimulus, reflecting a possible change in function [33]. We therefore were interested in these proteins as sensitive markers of toxicity in the lung and whether they would be altered after TS exposure. The involvement of p38 as a key signalling mediator in the response to TS has been examined previously in this model [22,34] and p-p38 was considered a suitable marker to reflect the signalling activity of the cells involved.

**Figure 5:**
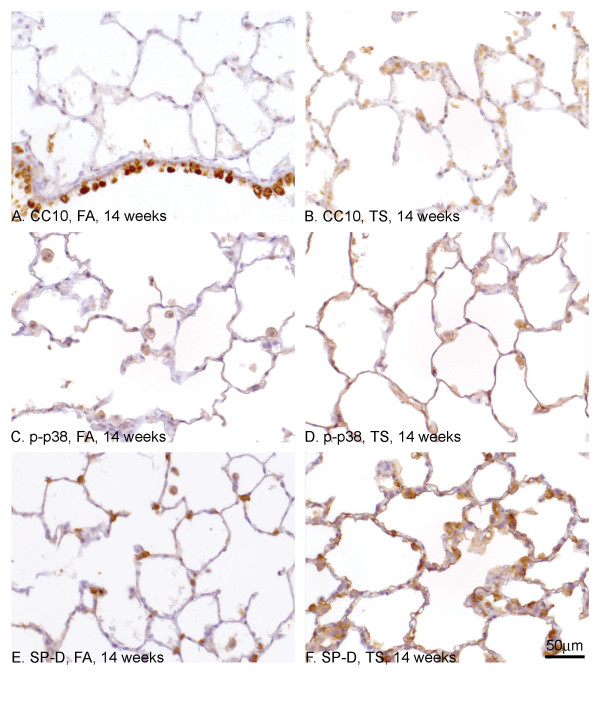
**Molecular profile of functional markers in the lung parenchyma of TS induced SH rats**. CC10 (A, B), p-p38 (C, D) and SP-D (E, F) expression were examined in the lung parenchyma after FA (A, C, E) and TS (B, D, F) exposure. After FA exposure, CC10 was only seen in the parenchyma in occasional alveolar macrophages and type II pneumocytes. The image shown in A includes a transitional airway to demonstrate normal Clara cell staining. After TS exposure, CC10 was now seen more diffusely in the alveolar bed in type II cells and alveolar macrophages (B). p-p38 (C) and SP-D (E) were observed in macrophages and type II pneumocytes within the alveolar bed in FA exposed SH rats and staining pattern appeared more extensive after TS exposure and included type I pneumocytes (D, F).

**Figure 6:**
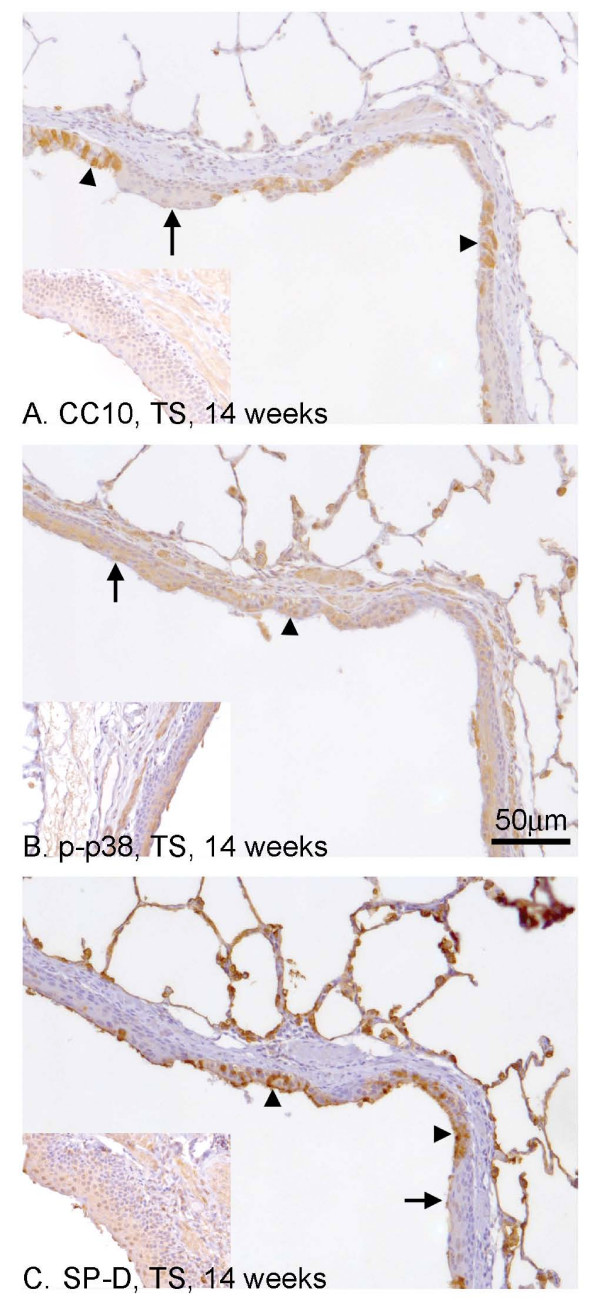
**Association of functional markers with squamous metaplasia in distal airways**. Antibodies against CC10 (A), p-p38 (B) and SP-D (C) were used to assess the effect of squamous metaplasia on epithelial cell functional markers. Within distal airways, there were areas of squamous metaplasia (arrow) contiguous with areas of non-squamous epithelial cells (arrowhead). CC10 (A) was lost from areas of squamous metaplasia (A, arrow), including the proximal airways (inset) but not from other areas of non-squamous airways (A, arrowhead). p-p38 (B, distal airway) was detected in all epithelial cells regardless of cell type including squamous metaplasia in the proximal airways (inset). SP-D (C) was lost from squamous regions in distal (C, arrow) and proximal airways (C, inset). In areas of non-squamous epithelium in the distal airways, epithelial cells were seen to stain positive (C, arrowhead).

We looked at the expression of CC10, p-p38 and SP-D as markers of epithelial function. CC10 staining was observed in the cytoplasm of Clara cells in all sizes of airways in FA exposed rats (see Fig. 5A for an example). This staining was lost in the squamous epithelium following 14 weeks TS exposure (Fig. 6A, distal airways, arrow. Inset shows proximal airways) but not from contiguous non-squamous epithelium (Fig. 6A, arrowhead). The non-squamous epithelium that was adjacent to the squamous epithelium showed similar staining patterns to the FA control rats (data not shown). In contrast, p-p38 was detected in the cytoplasm of both non-squamous epithelium (Fig. 6B, arrow) and squamous metaplasia cells (Fig. 6B, arrowhead and inset). Antibodies to SP-D appeared to stain weakly squamous metaplasia found in the proximal airways (Fig. 6C, inset), and the less stratified squamous metaplasia found in the distal airways (Fig. 6C, arrow). In non-squamous epithelium that was adjacent to the squamous epithelium(Fig. 6C, arrowhead), the staining pattern was similar to FA exposed rats (data not shown).

### Molecular profile of functional markers in the lung parenchyma following TS exposure

Following TS exposure, the parenchyma showed focal accumulations of inflammatory cells, particularly alveolar macrophages and foam cells in conjunction other changes to the alveolar bed (see Fig. 2A-D). Anti-CC10 antibodies were seen to stain type II pneumocytes in FA exposed rats (Fig. 5A). After TS exposure there were increased numbers of cells staining due to the increased cellularity within the alveolar bed (Fig. 2B). Anti-p-p38 antibodies were seen to stain alveolar macrophages and occasional type II pneumocytes in FA exposed rats (Fig. 5C). After TS exposure, the staining appeared more diffuse due to increased cellularity and type I pneumocytes also appeared to be staining positive along with alveolar macrophages and type II pneumocytes (Fig. 5D). Similar to p-p38, anti-SP-D antibodies were seen to stain type II pneumocytes and alveolar macrophages in FA exposed rats (Fig. 5E) and the staining pattern after TS exposure was much more diffuse with additional type I pneumocytes showing positive staining (Fig. 5F). In all 3 cases, the staining pattern after TS exposure was more diffuse due to the increased cellularity of the alveolar bed. The microvasculature within the alveolar bed appeared undamaged as assessed by anti-CD31 staining (data not shown).

### Comparison of squamous metaplasia in human COPD and TS exposed SH rat lungs

**Figure 7:**
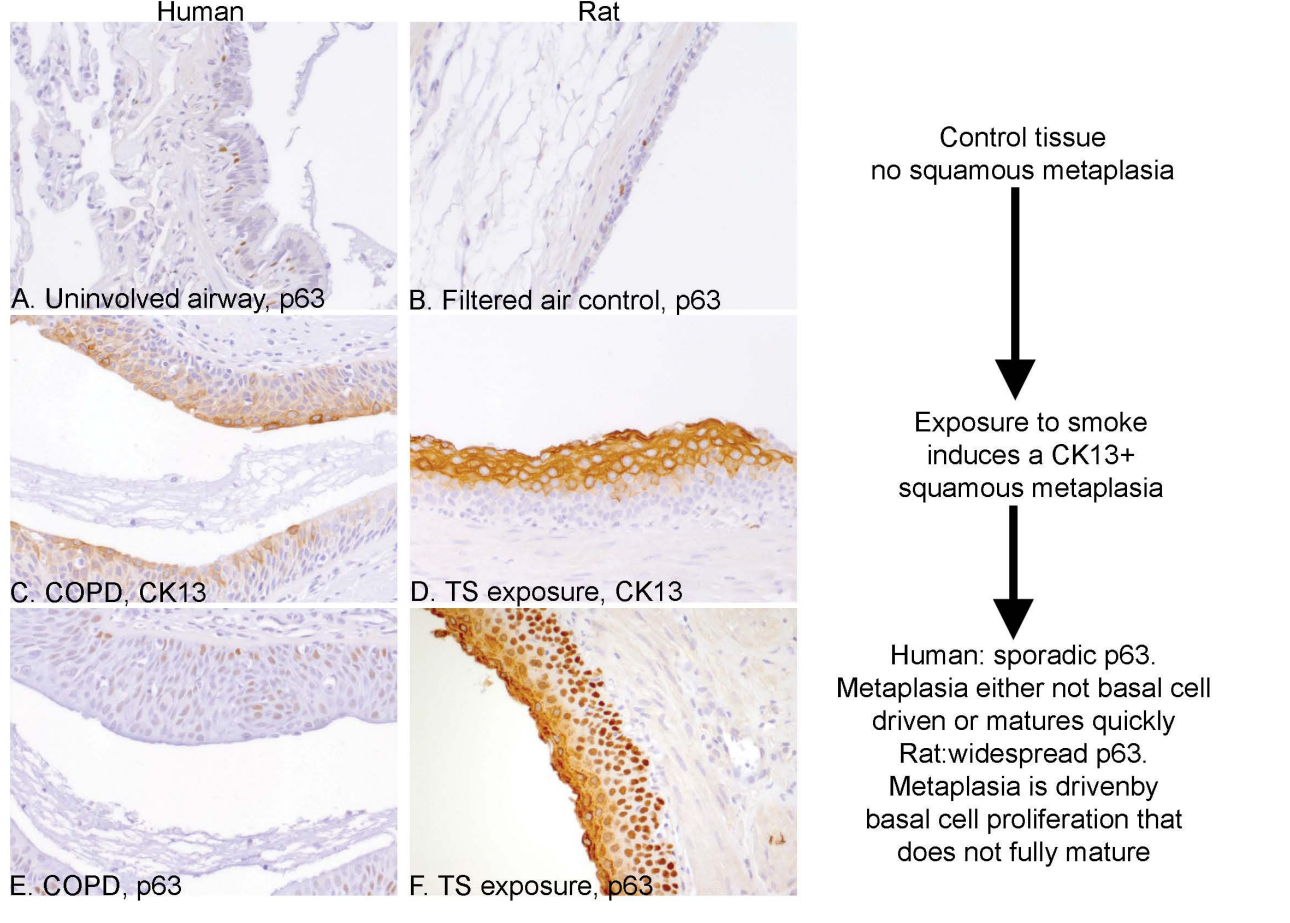
**Comparison of squamous metaplasia in human COPD and TS exposed SH rat lungs**. Squamous metaplasia was identified in human COPD lung tissue (C, E) and 14 week TS exposed SH rat lungs (D, F) and compared to either non-squamous airways in COPD lung (A) or FA exposed SH rats (B). They were stained for p63 (A, B, E, F) and CK13 (C, D). In the control non-squamous airways from both human (A) and rat (B), p63 stained occasional flattened cells close to the basement matrix of proximal airways. Areas of squamous metaplasia were identified in both human (C) and rat (D) tissue by CK13 staining. In contrast to the extensive p63 staining seen in the rat tissue (F), the human tissue showed only sporadic cells scattered through out the squamous metaplasia (E).

A comparison was made of the CK13 and the p63 profile in areas of squamous metaplasia from human COPD lung and TS exposed SH rat lungs. In human COPD lung, the areas of squamous metaplasia within the airway were focal, and unlike the rats in this study, it rarely encompassed the entire airway. For control purposes, airways with squamous metaplasia were compared with non-squamous airways from the same section (Fig. 7A, control). In humans, p63 is present in basal cells regardless of airway size (Fig. 7A, arrow). This is in contrast to the SH rats used in this study where, in non-squamous regions, p63-positive basal cells were only seen in the largest airways (Fig. 7B, arrow). Areas of squamous metaplasia were characterised by positive CK13 staining in both the human COPD tissue (Fig. 7C) and the TS exposed SH rats (Fig. 7D). This staining was largely confined to the parabasal and superficial layers of the squamous cells although in the human tissue, some staining was occasionally also seen in the most basal layer. In a marked contrast, the p63 staining in human squamous metaplasia highlighted sporadic cell nuclei scattered throughout the basal and parabasal layers (Fig. 7E), whereas the TS exposed SH rats, p63 staining was seen in all basal cells and in most parabasal cells in the region of metaplasia with a graded intensity the decreased away from the basement membrane (Fig. 7F).

## Discussion

COPD is a multi-factorial disease and existing animal models involving a single insult in an otherwise healthy animal are therefore limited in providing analogous experimental pathology. Based on our observations, the development and use of more complex, polygenic models such as the SH rat is likely to provide a more relevant model of human COPD. The similarities of the SH rat model with COPD are two fold. Firstly, the squamous metaplasia is found in the larger, more proximal airways of the SH rats exposed to TS, which is a similar anatomical location to human COPD lungs [3,35]. Secondly, the SH rats show borderline hypertension like many COPD patients although the mechanisms may differ [36]. Non-SH rats subject to chronic regimes of TS do not develop this pathology [19,37] indicating that the hypertensive nature of the rat is contributing to the development of the squamous metaplasia. Although our results indicate that the common pathology may arise via a different mechanism, and this may just be a reflection of inherent differences between rat and human, there is still considerable value in the utility of this rat stain for respiratory research. The SH rat is known to be susceptible to various inhaled toxicants such as sulphur dioxide [24] or residual oil fly ash [37] but the exact physiological reason for this is not fully understood. It is known that these rats have a defect in CD36, which leads to defects in fatty acid metabolism but does not affect the hypertension [26], but the exact impact this reduction in CD36 has on respiratory function is not yet known. SH rats also show an increased inflammatory response and mucus production following sulphur dioxide exposure compared to Sprague-Dawley rats and this may reflect how a compromised immune function can contribute to susceptibility to airway disease [24]. In addition to the borderline hypertension, the pulmonary vascular bed is known to be compromised in SH rats [37] and consistent with this, we found that microvascular leakage was prominent following TS exposure but the capillary bed still appeared patent as judged by CD31 staining. Another consequence of hypertension is hypoxia but this has not been explored in this model. It is likely that raised alveolar bed pressure could lead to local and regional hypoxia that also affects the larger airways via perturbation of the microvascular plexus that supplies the larger vessels and bronchioles. Further studies would be needed to investigate the contribution of hypoxia to the increased susceptibility and development of airways disease of the SH rat.

The squamous metaplasia appears to progress down the tracheobronchial tree with time and we have not been able to establish the mechanism behind this. There are two possible explanations. It could be due to the pathological changes spreading down the airways from an initial site of origin within the proximal airways. Alternatively, it could reflect a difference in the proliferation and development times between proximal and distal airways i.e. the pathology just takes longer to develop in bronchioles rather than bronchi. The profiles seen with CK13 (mature squamous cell marker), p63 (basal cell marker) and Ki-67 (proliferative marker) indicate that the squamous metaplasia is arising from the p63+, CK13- basal cells that proliferate and mature to form a CK13+ p63- barrier of squamous epithelium. The graded intensity of the p63 staining seen in the airways (Fig. 4F, strongest in cells closest to the basement membrane) could be reflection of the gradual loss of this basal marker as the epithelium matures. We chose these markers for our immunohistochemistry studies as western blot studies on whole lung homogenate from the same rats by Zhong and co-workers [22] have already shown that there was an increase CK5 (basal cell marker), which correlates with our p63 finding. Interestingly, the authors found there was a decrease in the markers of late epithelial maturation - loricrin, involucrin and filaggrin. This, coupled with our observation of an increase in CK13 indicates that the basal cells are maturing to a squamous epithelium but do not express markers of the terminally differentiated, mature epithelium. CK13 staining has also been shown to correlate with well-differentiated squamous cell metaplasia in human tissue [9,38]. The situation in rat is less clear where Kal and co-workers [11] showed CK13 reactivity in 1 out of 3 well differentiated squamous cell carcinomas. The CK profiles have also been examined in the upper and lower respiratory tract following TS exposure (Wistar rats, 12 hrs/day, 5 days/week, 8 weeks) but found neither pathological changes in the lungs nor any CK13 reactivity [39]. However, their data were generated in Wistar rats and not SH rats. Our data indicate that chronic TS exposure in SH rats can lead to the development of a squamous metaplasia that is both CK13+ and keratinised in the upper airways that potentially arises from the basal cells. When compared to human pulmonary squamous metaplasia, this suggests key differences in the progenitor cell populations and the mechanics of epithelial repair in rat lungs compared to human lungs. However, we cannot rule out the possibility that due to differences in cell turnover kinetics between rat and man, the lesions are of essentially the same phenotype - albeit at different stages of maturation. However, we would suggest that the distinct compartmentalisation of p63 and CK13 expression in the rat supports the case for a distinct mucosal remodelling phenotype compared to man.

The presence of the squamous metaplasia in the proximal airways also had a potential functional consequence. There was a loss of staining in areas of squamous metaplasia for the epithelial proteins CC10 and surfactant D, which are required for airway epithelial cell function. This could indicate a change in function from secretory airway epithelial cells, presumably to a more barrier-type function, although we have not applied any specific markers to investigate this. A lack of staining for CC10 has also been shown in samples of squamous metaplasia in human bronchi [38] and in mice, the levels of CC10 in tumours may also be indicative of the state of tumour progression as larger carcinomas showed consistently less staining [40]. Also, Stripp and colleagues have demonstrated that CC10 expressing (CE) cells, including the variant CE, are important stem cells in bronchial epithelium in mice but when they are lost, an alternative basal cell will then serve as a progenitor cell [41,42]. Our studies are in accordance with these results as we have demonstrated a loss of CC10 but an increase in p63+ basal cells.

Conversely, there was an increase in the numbers of cells staining for CC10 and surfactant D in the periphery of the TS exposed lungs particularly type II pneumocyte around affected bronchioles. The more diffuse staining pattern appears to be from an increase in the numbers of resident cells (mainly type II pneumocytes) and from infiltration of inflammatory cells but also the presence of staining in new cell populations. Type I pneumocytes now showed staining for p-p38 and surfactant D. The result for p-p38 is in agreement with the complementary study to ours performed by Zhong and co-workers [22], who showed by Western blotting that there was a 3-fold increase in p-p38. We have added to this information by further defining the parenchyma as the compartment in which this occurs. We have previously shown similar results in a repeat LPS challenge model [33] and we hypothesise that this altered profile for proteins such as CC10 and surfactant D in the alveolar bed is an attempt by the lung to compensate for losses in the proximal airways and to maintain airway patency.

Within the peripheral alveolar bed compartment of TS exposed SH rats, a number of pathological changes are seen which are consistent with human COPD lung including presence of mononuclear inflammatory cells, matrix deposition and remodelling of the alveolar bed, parenchymal airspace enlargement and a loss of connectivity. We have chosen to use the term airspace enlargement rather than emphysema due to the inherent hyperinflation seen in rodents, which is often described as emphysema and is considered as a pathological consequence of the particular insult being examined. It is not known whether the damage and remodelling in the alveolar bed is directly due to the presence of inflammatory cells seen within the alveolar bed or whether it is a direct effect of the TS on the resident cells. This study showed a marginal increase in activated caspase 3 by immunohistochemistry and the associated study by Zhong and co-workers showed an increase, by Western blotting, in the parenchyma of activated caspases 3, 8 and 9 [29]. This indicates that loss of resident structural cells via apoptosis could also be a mechanism that contributes to airspace enlargement. Smith and co-workers [43] used the same model to show that a catalytic antioxidant could reduce the numbers of cells in the bronchoalveolar lavage fluid and reduce pathological changes associated with TS exposure indicating that it is the trafficking of the inflammatory cells that is playing an important role. The results indicate that chronic exposure to TS induces changes in the alveolar bed compartment and further characterisation and morphometric analysis of the distal airway and alveolar bed changes are to be the focus of a subsequent paper (Bolton et al., manuscript in preparation).

This study also highlights some intriguing differences between rat and human airways that have not been documented before. Immunostaining for p63 was seen in the basal cells of human airways in nearly all airways seen regardless of size. However, in the rats, p63+ basal cells were only seen in the larger airways. This was not due to the lack of basal cells in smaller airways, as some basal cells could be identified next to the basement membrane on an H&E stained section. This suggests that the progenitor cell population is different in rats versus humans, particularly in the distal airways. The p63 staining in rat squamous metaplasia also showed a graded intensity that decreased away from the basement membrane indicating that the basal cells are maturing and losing their p63 staining as they become squamous. Despite this, the pathology described in this paper is centred on the comparison of the larger airways that have a similar p63 profile. We have shown in humans that the p63-positive cell population is sporadic compared to rat, indicating that in humans the basal cells are either not the key drivers of the squamous metaplasia or that their expression is lost very rapidly and epithelium matures more quickly. Consistent with this is a report that p63 and CK14 levels are increased in a rat multi-layered epithelium model of Barrett's esophagus compared to human tissue samples [44]. Involucrin, a marker of late epithelial maturation, is also increased in COPD [6], suggesting that the squamous metaplasia matures to a greater extent in humans than in rats. These results highlight key differences at a molecular level between a morphologically similar squamous metaplasia in humans and rats. Further studies would be needed to elucidate whether this was a reflection of the reversibility of the pathology as the squamous metaplasia seen in this SH rat study will revert with time (KE Pinkerton, unpublished observations), but full reversion is unlikely in humans [3,36]. It should be noted that the conditions of the experimental procedures in rats are tightly controlled whereas human behaviour and smoking patterns are not and can have a profound influence on the pathology of the disease.

## Conclusion

We have demonstrated that exposure of SH rats to a chronic regime of TS results in a squamous metaplasia within the proximal airways that has functional effects in both the proximal and the distal airways. The squamous metaplasia was shown to have morphological similarities to lesions found in human COPD. However, there were key differences at the molecular level, particularly of the basal cells, that highlight potential differences in the mechanism by which the metaplasia arises. We also speculate whether the progenitor cell populations within the lung are different in rats compared to humans.

## Abbreviations

CC10: Clara Cell 10 kDa Protein; COPD: Chronic Obstructive Pulmonary Disease; CK: Cytokeratin; H&E: Haematoxylin and Eosin; LPS: Lipopolysaccharide; SH: spontaneous hypertensive; SP-D: surfactant protein D; TS: tobacco smoke.

## Competing interests

The authors declare that they have no competing interests.

## Authors' contributions

SB and KP carried out all the immunohistochemical work. SB drafted the manuscript. VO, MF and KEP participated in the design of the study, provided discussion and helped draft the manuscript. The SH rat TS exposure work was carried out in the lab of KEP.

## References

[B1] Hogg JC Pathophysiology of airflow limitation in chronic obstructive pulmonary disease The Lancet 2004 364 709 721 10.1016/S0140-6736(04)16900-6 15325838

[B2] Kim V Rogers TJ Criner GJ New Concepts in the Pathobiology of Chronic Obstructive Pulmonary Disease Proc Am Thorac Soc 2008 5 478 485 18453359 10.1513/pats.200802-014ET PMC2645323

[B3] Lapperre T Sont J van Schadewijk A Gosman M Postma D Bajema I Timens W Mauad T Hiemstra P the GLUCOLD Study Group Smoking cessation and bronchial epithelial remodelling in COPD: a cross-sectional study Resp Res 2007 8 85 10.1186/1465-9921-8-85 PMC2214729 18039368

[B4] Puchelle E Zahm JM Tournier JM Coraux C Airway Epithelial Repair, Regeneration, and Remodeling after Injury in Chronic Obstructive Pulmonary Disease Proc Am Thorac Soc 2006 3 726 733 10.1513/pats.200605-126SF 17065381

[B5] Rennard SI Inflammation and Repair Processes in Chronic Obstructive Pulmonary Disease Am J Respir Crit Care Med 1999 160 12S 16 10.1164/ajrccm.160.supplement_1.5 10556162

[B6] Araya J Cambier S Markovics JA Wolters P Jablons D Hill A Finkbeiner W Jones K Broaddus VC Sheppard D Squamous metaplasia amplifies pathologic epithelial-mesenchymal interactions in COPD patients J Clin Invest 2007 117 3551 3562 17965775 10.1172/JCI32526 PMC2040320

[B7] Broers JLV de Leij L Klein Rot M ter Haar A Lane B Leigh IM Wagenaar S Vooijs GP Ramaekers FCS Expression of intermediate filament proteins in fetal and adult human lung tissues Differentiation 1989 40 119 128 10.1111/j.1432-0436.1989.tb00821.x 2474472

[B8] Kasper M Rudolf T Verhofstad AAJ Schuh D Muller M Heterogeneity in the immunolocalization of cytokeratin-specific monoclonal antibodies in the rat lung: evaluation of three different alveolar epithelial cell types Histochem 1993 100 65 71 10.1007/BF00268879 7693628

[B9] Broers JLV Ramaekers FCS Rot MK Oostendorp T Huysmans A van Muijen GNP Wagenaar SS Vooijs GP Cytokeratins in Different Types of Human Lung Cancer as Monitored by Chain-specific Monoclonal Antibodies Cancer Res 1988 48 3221 3229 2452687

[B10] Schlage WK Bulles H Friedrichs D Kuhn M Teredesai A Cytokeratin expression patterns in the rat respiratory tract as markers of epithelial differentiation in inhalation toxicology. I. Determination of normal cytokeratin expressino patterns in nose, larynx, trachea and lung Toxicol Pathol 1998 26 324 343 10.1177/019262339802600307 9608639

[B11] Kal HB van Berkel AH Broers JLV Klein JC Mijnheere EP Roholl PJM Ramaekers FCS Cytokeratins expressed in experimental rat bronchial carcinomas Int J Cancer 1993 53 506 513 10.1002/ijc.2910530325 7679092

[B12] Weinstein MH Signoretti S Loda M Diagnostic Utility of Immunohistochemical Staining for p63, a Sensitive Marker of Prostatic Basal Cells Mod Pathol 2002 15 1302 1308 10.1097/01.MP.0000038460.95912.6E 12481011

[B13] Barbieri CE Pietenpol JA p63 and epithelial biology Exp Cell Res 2006 312 695 706 10.1016/j.yexcr.2005.11.028 16406339

[B14] Murray-Zmijewski F Lane DP Bourdon JC p53//p63//p73 isoforms: an orchestra of isoforms to harmonise cell differentiation and response to stress Cell Death Differ 2006 13 962 972 10.1038/sj.cdd.4401914 16601753

[B15] Chilosi M Poletti V Murer B Lestani M Cancellieri A Montagna L Piccoli P Cangi G Semenzato G Doglioni C Abnormal re-epithelialization and lung remodelling in idiopathic pulmonary fibrosis: the role of dN-p63 Lab Invest 2002 82 1335 1345 12379768 10.1097/01.lab.0000032380.82232.67

[B16] Hibi K Trink B Patturajan M Westra WH Caballero OL Hill DE Ratovitski EA Jen J Sidransky D AIS is an oncogene amplified in squamous cell carcinoma Proc Natl Acad Sci USA 2000 97 5462 5467 10805802 10.1073/pnas.97.10.5462 PMC25851

[B17] Senoo M Tsuchiya I Matsumura Y Mori T Saito Y Kato H Okamoto T Habu S Transcriptional dysregulation of the *p73L*/*p63*/*p51*/*p40*/*KET *gene in human squamous cell carcinomas: expression of Np73L, a novel dominant-negative isoform, and loss of expression of the potential tumour suppressor p51 Br J Cancer 2001 84 1235 1241 11336476 10.1054/bjoc.2000.1735 PMC2363892

[B18] Haschek AM Rousseaux CG Fundamentals of Toxicologic Pathology 1998 Academic Press

[B19] Hahn FF Gigliotti AP Hutt JA March TH Mauderly JL A review of the histopathology of cigarette smoke-induced lung cancer in rats and mice Int J Toxicol 2007 26 307 313 10.1080/10915810701483450 17661221

[B20] Walker NJ Yoshizawa K Miller RA Brix AE Sells DM Jokinen MP Wyde ME Easterling M Nyska A Pulmonary lesions in female Harlan Sprague-Dawley rats following two year oral treatment with dioxin-like compounds Toxicol Pathol 2007 35 880 889 18098034 10.1080/01926230701748396 PMC2633090

[B21] Mohr U Ernst H Roller M Pott F Pulmonary tumor types induced in Wistar rats of the so-called "19-dust study" Exp Toxicol Path 2006 58 13 20 10.1016/j.etp.2006.06.001 16806863

[B22] Zhong CY Zhou YM Douglas GC Witschi H Pinkerton KE MAPK/AP-1 signal pathway in tobacco smoke-induced cell proliferation and squamous metaplasia in the lungs of rats Carcinogenesis 2005 26 2187 2195 10.1093/carcin/bgi189 16051644

[B23] Kodavanti UP Costa DL Rodent models of susceptibility: what is their place in inhalation toxicology? Resp Physiol 2001 128 57 70 10.1016/S0034-5687(01)00265-1 11535263

[B24] Kodavanti UP Schladweiler MC Ledbetter AD Ortuno RV Suffia M Evansky P Richards JH Jaskot RH Thomas R Karoly E The Spontaneously Hypertensive Rat: An Experimental Model of Sulfur Dioxide-Induced Airways Disease Toxicol Sci 2006 94 193 205 10.1093/toxsci/kfl087 16929007

[B25] Yu B Kodavanti UP Takeuchi M Witschi H Pinkerton KE Acute Tobacco Smoke-Induced Airways Inflammation in Spontaneously Hypertensive Rats Inhal Toxicol 2008 20 623 633 10.1080/08958370701861538 18464051

[B26] Pravenec M Landa V Zídek V Musilová A Kazdová L Qi N Wang J St Lezin E Kurtz T Transgenic expression of CD36 in the spontaneously hypertensive rat is associated with amelioration of metabolic disturbances but has no effect on hypertension Physiol Res 2003 52 681 688 14640889

[B27] Vaughan P Waller DA Surgical treatment of pulmonary emphysema Surgery (Oxford) 2005 23 435 438 10.1383/surg.2005.23.12.435

[B28] Wilson E Jackson S Cruwys S Kerry P An evaluation of the immunohistochemistry benefits of boric acid antigen retrieval on rat decalcified joint tissues J Immunol Meth 2007 322 137 142 10.1016/j.jim.2007.01.020 17362980

[B29] Zhong CY Zhou YM Pinkerton KE NF-[kappa]B inhibition is involved in tobacco smoke-induced apoptosis in the lungs of rats Toxicol Appl Pharmacol 2008 230 150 158 18355884 10.1016/j.taap.2008.02.005 PMC2495769

[B30] Hermans C Knoops B Wiedig M Arsalane K Toubeau G Falmagne P Bernard A Clara cell protein as a marker of Clara cell damage and bronchoalveolar blood barrier permeability Eur Respir J 1999 13 1014 1021 10.1034/j.1399-3003.1999.13e14.x 10414398

[B31] Pinkerton KE Dodge DE Cederdahl-Demmler J Wong VJ Peake J Haselton CJ Mellick PW Singh G Plopper CG Differentiated bronchiolar epithelium in alveolar ducts of rats exposed to ozone for 20 months Am J Pathol 1993 142 947 956 8456949 PMC1886801

[B32] Van Miert E Dumont X Bernard A CC16 as a amarker of lung epithelial hyperpermeability in an acute model of rats exposed to mainstream cigarette smoke Toxicol Letts 2005 159 115 123 10.1016/j.toxlet.2005.05.007 16165332

[B33] Bolton SJ Pinnion K Marshall CV Wilson E Barker J Oreffo V Foster ML Changes in Clara Cell 10 kDa Protein (CC10)-positive cell distribution in acute lung injury following repeated lipopolysaccharide challenge in the rat Toxicol Pathol 2008 36 440 448 10.1177/0192623308315357 18420837

[B34] Zhong CY Zhou YM Pinkerton KE NF-[kappa]B inhibition is involved in tobacco smoke-induced apoptosis in the lungs of rats Toxicol Appl Pharmacol 2008 230 150 158 18355884 10.1016/j.taap.2008.02.005 PMC2495769

[B35] Papi A Casoni G Caramori G Guzzinati I Boschetto P Ravenna F Calia N Petruzzelli S Corbetta L Cavallesco G COPD increases the risk of squamous histological subtype in smokers who develop non-small cell lung carcinoma Thorax 2004 59 679 681 15282388 10.1136/thx.2003.018291 PMC1747095

[B36] Rennard SI Clinical Approach to Patients with Chronic Obstructive Pulmonary Disease and Cardiovascular Disease Proc Am Thorac Soc 2005 2 94 100 10.1513/pats.200410-051SF 16113475

[B37] Kodavanti UP Schladweiler MC Ledbetter AD Watkinson WP Campen MJ Winsett DW Richards JR Crissman KM Hatch GE Costa DL The Spontaneously Hypertensive Rat as a Model of Human Cardiovascular Disease: Evidence of Exacerbated Cardiopulmonary Injury and Oxidative Stress from Inhaled Emission Particulate Matter Toxicol Appl Pharmacol 2000 164 250 263 10.1006/taap.2000.8899 10799335

[B38] Barth P Koch S Muller B Unterstab F von Wichert P Moll R Proliferation and number of Clara cell 10 kDa protein (CC10)-reactive epithelial cells and basal cells in normal, hyperplastic and metaplastic bronchial mucosa Virchows Arch 2000 437 648 655 10.1007/s004280000316 11193477

[B39] Schlage WK Bulles H Friedrichs D Kuhn M Teredesai A Terpstra PM Cytokeratin expression patterns in the rat respiratory tract as markers of epithelial differentiation in inhalation toxicology. II. Changes in cytokeratin expression patterns following 8 day exposure to room-aged cigarette sidestream smoke Toxicol Pathol 1998 26 344 360 10.1177/019262339802600308 9608640

[B40] Wikenheiser KA Whitsett JA Tumor progression and cellular differentiation of pulmonary adenocarcinomas in SV40 large T antigen transgenic mice Am J Respir Cell Mol Biol 1997 16 713 723 9191473 10.1165/ajrcmb.16.6.9191473

[B41] Hong KU Reynolds SD Watkins S Fuchs E Stripp BR Basal Cells Are a Multipotent Progenitor Capable of Renewing the Bronchial Epithelium Am J Pathol 2004 164 577 588 14742263 10.1016/S0002-9440(10)63147-1 PMC1602270

[B42] Hong KU Reynolds SD Giangreco A Hurley CM Stripp BR Clara Cell Secretory Protein-Expressing Cells of the Airway Neuroepithelial Body Microenvironment Include a Label-Retaining Subset and Are Critical for Epithelial Renewal after Progenitor Cell Depletion Am J Respir Cell Mol Biol 2001 24 671 681 11415931 10.1165/ajrcmb.24.6.4498

[B43] Smith KR Uyeminami D Kodavanti UP Crapo JR Chang L-Y Pinkerton KE Inhibition of tobacco smoke-induced lung inflammation by a catalytic antioxidant Free Rad Biol Med 2002 33 1106 1114 10.1016/S0891-5849(02)01003-1 12374622

[B44] Chen X Qin R Liu B Ma Y Su Y Yang CS Glickman JN Odze RD Shaheen NJ Multilayered epithelium in a rat model and human Barrett's esophagus: similar expression patterns of transcription factors and differentiation markers BMC Gastroenterol 2008 8 1 18190713 10.1186/1471-230X-8-1 PMC2267197

